# Strongyloidiasis in humans and dogs in Southern Italy: an observational study

**DOI:** 10.1007/s00436-023-07978-1

**Published:** 2023-09-22

**Authors:** Paola Paradies, Serena Digiaro, Antonella Colella, Beatrice Greco, Alessandra Recchia, Marco Giuseppe Prato, Cristina Mazzi, Giuseppe Losurdo, Alfredo Di Leo, Fabio Formenti, Dora Buonfrate

**Affiliations:** 1 Department of Precision and Regenerative Medicine and Ionian Area (DiMePRe-J), University of Bari “Aldo Moro”, Valenzano, Bari, Italy; 2 Department of Infectious, Tropical Diseases and Microbiology, IRCCS Sacro Cuore Don Calabria Hospital, Negrar, Verona, Italy; 3 Clinical Research Unit, IRCCS Sacro Cuore Don Calabria Hospital, Negrar, Verona, Italy

**Keywords:** *Strongyloides stercoralis*, Dogs, Humans, ELISA, RT-PCR

## Abstract

Strongyloidiasis is a clinical issue both in humans and in dogs. Moreover, there are concerns about its zoonotic potential. We aimed to explore *Strongyloides stercoralis* epidemiology in Southern Italy in humans and dogs sharing the same environment in three different settings: (1) kennels (group K); (2) livestock farms (group L) and (3) agricultural farms (group A). For humans, a commercial ELISA test was used for screening. RT-PCR on faecal samples was done for people testing positive or equivocal at serology. On dog’s faecal samples, Baermann test and RT-PCR were performed. A total of 145 dogs and 139 persons were tested. Based on faecal tests in dogs and serology in humans, a *S. stercoralis* positivity of 4.1% and 6.5% was revealed, respectively. The sites where cases were found were different for animals and humans. In dogs the highest positivity was in group K (6.7% against 2% and 0% in L and A). Differently, in humans the proportion of positive results was similar between the groups (*p* = 0.883). Fifty percent (3/6) of positive dogs were healthy; the other dogs presented weight loss and/or diarrhoea. ELISA-positive persons (*n*=9) were all in health, but abdominal pain (37.5%), urticaria (22.2%) and asthma (22.2%) were reported, resolving after treatment with oral ivermectin 200 μg/kg. RT-PCR performed on 13 human faecal samples resulted negative. These findings suggest that strongyloidiasis is present in humans and dogs in Southern Italy, and screening in larger cohorts would be needed for more accurate estimates.

## Background

*Strongyloides stercoralis* is a soil-transmitted nematode with clinical and epidemiological relevance in humans, due to its high prevalence and its capacity to induce a life-threatening hyperinfection (Nutman 2017).

*S. stercoralis* is widely present in tropical and subtropical countries; occasionally, cases are diagnosed in temperate countries both in humans and in dogs (Ottino et al. 2020).

Sources of infection are filariform larvae present in soil contaminated by infected faeces; the larvae penetrate through the skin. In humans, after the first life cycle, a process of autoinfection begins, which persists indefinitely in the host if the infection is not effectively treated. The infection can remain asymptomatic for many years or forever. When present, symptoms mainly affect the skin, the abdomen, and the respiratory tract. Immunosuppression can cause disseminated strongyloidiasis, which is a massive and almost invariably fatal invasion of virtually all organs and tissues.

Canine infection can be asymptomatic or induce mild to severe disease, mostly associated to gastroenteric involvement (Dillard et al. 2007; Paradies et al. 2017), but respiratory and cutaneous signs have also been described (Cervone et al. 2016; Dashchenko et al. 2020). Some dogs can develop severe disease, even leading to death; therefore, a correct diagnosis in clinical practice is essential, especially since the commonly used deworming drugs for dogs (i.e. pyrantel, febantel) seem to be ineffective against *S. stercoralis* (Paradies et al. 2019).

A few studies provided evidence about the zoonotic potential of *S. stercoralis*, but the transmission from dogs to humans remains uncertain (Jaleta et al. 2017; Nagayasu et al. 2017; Beknazarova et al. 2019).

Aim of the present work was to assess the extent of *S. stercoralis* infection in Southern Italy in humans and dogs sharing the same environment.

## Material and methods

The study was conducted in Southern Italy (Apulia Region). Dogs and humans to be included in the study and sampled were selected from three different settings:

(1) Group K: kennels/shelters: dogs, workers and veterinarians; (2) group L: livestock farms: farm dogs, workers and owners and (3) group A: agricultural farms: farm dogs (including known and friendly free-living dogs) and workers. People and dogs living in the same farm or kennel were sampled on the same day. Medical history was registered for each dog. Informed consent was signed by the owner or the kennel manager before including the dog in the study. Faecal samples were collected directly from ampulla. In each farm, all approachable dogs were sampled, while in each kennel, ten dogs were randomly selected and sampled.

People aged >50 years of both sex and sharing the same environment of sampled dogs (living and/or working in the same farm or kennel of sampled dogs) were included in the study. The age group was selected based on published data on the epidemiology of strongyloidiasis in northern Italy, demonstrating increasing prevalence with older ages (Buonfrate et al. 2016). A project code was assigned using the same criterion as for dogs. Age, sex and medical history were registered. An informed consent and a consent for privacy forms were signed before inclusion.

For humans, a commercial ELISA assay (*Strongyloides ratti* ELISA by Bordier Affinity Products SA, Switzerland) was used for screening. Blood samples were collected by finger pricking to obtain dried blood spots absorbed on filter papers, as previously described (Formenti et al. 2016). Filter papers were shipped to IRCCS Sacro Cuore Don Calabria Hospital, Verona, where the tests were run. To compare the results of the serology tests between runs, we calculated the normalised optical density as the signal to the cut-off ratio. An OD ratio ≥ 1 was considered positive. An OD ratio between 0.8 and 1 was considered equivocal. A faecal sample for real-time (RT-PCR) was collected at a later time from people resulted positive or equivocal at ELISA.

On dogs’ faecal samples, modified Baermann test was performed within 12 h from collection to detect moving larvae. For both humans (positive or equivocal to ELISA) and all dogs, 1 gr of faecal sample was stored in ethanol 70% (Becker et al. 2015) and shipped to Verona for molecular biology analysis. Stool specimens were treated as previously described (Formenti et al. 2015), with minor changes.

The RT-PCR was based on Verweji’s method (Verweij et al. 2009), used in routine practice. Positive and negative controls were included in each RT-PCR run. All reaction and data analysis were performed on the CFX96 platform (BioRad, Milan, Italy). For all the RT-PCR analysis, the threshold was set at 200. Samples with a Ct >= 40 were considered negative.

People resulted positive at the ELISA test, irrespective of the result of RT-PCR, were invited to undergo a medical examination and, in the absence of hindering factors, they were treated with a single oral dose of 200 μg/kg ivermectin (Buonfrate et al. 2019). Full blood count was performed before and 1 month after treatment. Positive dogs were treated as well with ivermectin per os (Paradies et al. 2019).

The study received the ethical approval for both human and veterinary aspects from the “Ethics Committee for Veterinary Clinical and Zootechnical Studies of the Department of Emergency and Organ Transplantation” Approval n. 04/2022 and “Indipendent Ethics Commitee Azienda Ospedaliero-Universitaria Consorziale Policlinico” n. 7396/22.

Demographic and clinical data were summarised using descriptive statistics and measures of variability. Continuous variables were summarised with median and interquartile ranges, while count variables were summarised with absolute and percentage frequencies. The chi-square test of homogeneity was used to compare the proportions of positive results within different groups. A *p* value of <0.05 was considered statistically significant.

## Results and discussion

**Fig. 1:**
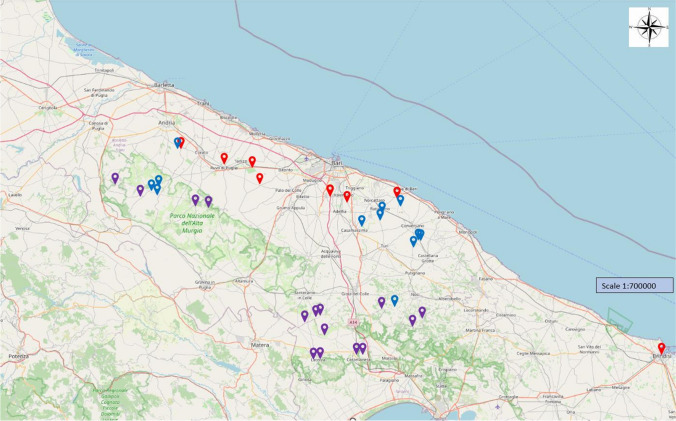
(MAP 1): Distribution of sampled farms and kennels differentiated by colours (red=kennels; violet=livestock farms; Blue=agricultural farms)

A total of 145 dogs and 139 persons were tested. The distribution of sampled farms/kennels is reported in map 1 (Fig. 1).

On the whole, 6/145 adult dogs (4.1%) resulted positive at faecal RT-PCR; in particular, 5/75 dogs of K group (6.7%) and 1/49 of L group (2%); no positives were found in group A. Of positive dogs from group K, three dogs were of the same kennel and the other two were from another. At the Baermann test, 4/145 samples resulted positive for *S. stercoralis* larvae. All dogs positive at RT-PCR had *S. stercoralis* larvae revealed at Baermann test, except for two (Baermann test failed to identify two positive dogs).

**Fig. 2:**
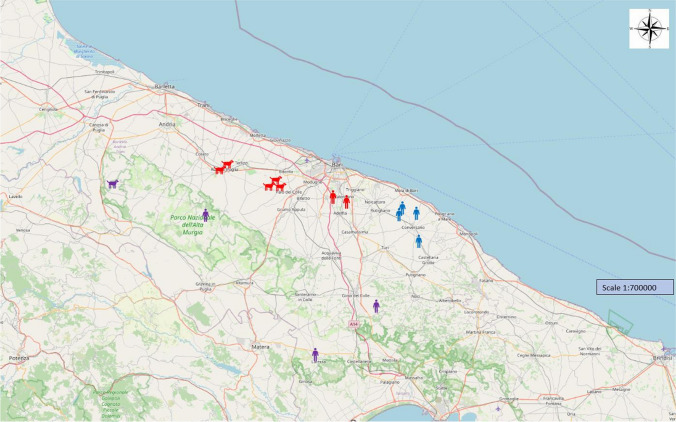
(MAP 2): Geographic distribution of positive dogs (*n*=6) and humans (*n*=9). Different colours as in map 1 are indicative of the different habits (red= kennels, blue= agricultural farms; violet= livestock farm). None of the kennels/farms positive for dogs were positive for humans and vice-versa

Nine out of the 139 persons (6.5%) had positive serology. The proportion of positive results was similar between the groups (*p* = 0.883); in particular, 2/35 persons resulted positive in group K (5.7%), 4/51 in group A (7.8%) and 3/53 in group L (5.7%). Geographical distribution of positive dogs and humans is reported in map 2 (Fig. 2). RT-PCR was performed on 13 human faecal samples (from nine ELISA-positive and four ELISA-equivocal persons); they all resulted negative. None of the positive dogs shared the same location (farm or kennel) with a positive person, to say that none of the farms or kennels where a positive dog was found had an ELISA-positive person and vice versa.

Briefly, the six positive dogs were four males and two females ranging from 2 to 10 years, five from two kennels and one from a livestock farm. Three out of the six positive dogs were healthy and no clinical signs were referred; the other three dogs had weight loss and/or diarrhoea.

ELISA-positive persons were five males and four females ranging from 51 to 64 years. Two were kennel workers, three were from livestock farms and four worked in agricultural farms. Abdominal pain was the most frequent symptom (three patients), which resolved after therapy in all patients. Two patients complained of urticaria, which totally resolved in one individual and improved in the other as referred at the clinical follow-up 6 months after treatment. Asthma was referred by two patients, with full clearance in one of them. The median eosinophil count was within normal ranges both at baseline and at follow-up, for all serology-positive people.

In this study, the screening approach differed between dogs and humans. The reason of this choice is that in humans because of autoinfection and possible hyperinfection, it is mandatory to deploy the highest sensitive test (serology) in order to limit the number of undetected cases. Conversely in dogs, serology was not considered the right approach due to the possible self-cure and the lack of data on antibody persistence. Besides, ELISA test in dogs showed very low sensibility compared to other tests (Iatta et al. 2019). In humans, none of the serology positive participants had either positive RT-PCR or eosinophilia. This might mean either that these people had a low parasitic load, or that we observed false-positive serology results. In any case, based on the possible harm caused by strongyloidiasis in humans who might get immunosuppressed any time in life, and the excellent safety profile of ivermectin (Hürlimann et al. 2023), it is recommended to treat even people with suspected, unconfirmed strongyloidiasis. Hence, we offered treatment to all participants testing positive to serology.

Dogs included in this study were tested with Baermann and RT-PCR. Iatta et al. (2019) suggested that the association of RT-PCR with Baermann test could be the best approach to detect both symptomatic and asymptomatic infection. In dogs, RT-PCR showed 100% specificity and a sensitivity higher than Baermann method (Iatta et al. 2019). The Baermann test failed to identify two positive dogs in this study. In clinical practice, false negative results using the Baermann test are not surprising, since the larval output is irregular and may be low (Siddiqui and Berk 2001).

It is interesting to note that all positive dogs, except for one, came from K group, suggesting that life in kennels represents a risk factor for the infection probably because of overcrowding in restricted areas, associated to not-ideal sanitary conditions. In fact, the two positive kennels were not breeding kennels, but dog shelters hosting caught free-roaming and stray dogs. Although we did not find positive workers from the same kennels in this study, this aspect needs reflection if we consider the zoonotic potential of *S. stercoralis* infection and the trend of moving pets for adoption.

In literature, sporadic canine infections have been described in Europe, with hot spots documented in Southern Italy (Paradies et al. 2017, 2019).

In humans, data on aggregated cases and epidemiological surveys in Europe reveal the presence of the infection in Italy, Spain, France, Poland, Austria, Slovakia, Romania and Turkey (Ottino et al. 2020). In Italy, a large case-control study contributed to outline the epidemiology of strongyloidiasis in the North (Buonfrate et al. 2016), while there are scattered data about southern and central regions.

One main limitation of this study is that all people tested with RT-PCR resulted negative. Negative RT-PCR does not rule out the infection, as its sensitivity for *S. stercoralis* is known to be unsatisfactory (Buonfrate et al. 2018); however, a positive test would have been useful to confirm the infection and to carry out genotyping analyses to compare human and dog worm species. Moreover, we screened a limited number of individuals in three settings; hence, these figures might not mirror the prevalence of strongyloidiasis in the general population living in these provinces.

In conclusion, these findings suggest that strongyloidiasis is present in humans and dogs in Southern Italy, and screening larger cohorts would be needed to give more accurate estimates. Unfortunately, the sites where cases were found were different for animals and humans, and an in-depth analysis of possible zoonotic transmission could not be done.

## Data Availability

The raw data are available in Zenodo: https://zenodo.org/record/8359641.

## Abbreviations

RT-PCR: real-time PCR
